# The effect of sociodemographic factors on COVID-19 incidence of 342 cities in China: a geographically weighted regression model analysis

**DOI:** 10.1186/s12879-021-06128-1

**Published:** 2021-05-07

**Authors:** Huihui Zhang, Yini Liu, Fangyao Chen, Baibing Mi, Lingxia Zeng, Leilei Pei

**Affiliations:** Department of Epidemiology and Health Statistics, School of Public Health, Xi’an Jiaotong University Health Science Center, Xi’an, Shaanxi 710061 People’s Republic of China

**Keywords:** COVID-19, Sociodemographic factor, Spatial modeling, Spatial distribution

## Abstract

**Background:**

Since December 2019, the coronavirus disease 2019 (COVID-19) has spread quickly among the population and brought a severe global impact. However, considerable geographical disparities in the distribution of COVID-19 incidence existed among different cities. In this study, we aimed to explore the effect of sociodemographic factors on COVID-19 incidence of 342 cities in China from a geographic perspective.

**Methods:**

Official surveillance data about the COVID-19 and sociodemographic information in China’s 342 cities were collected. Local geographically weighted Poisson regression (GWPR) model and traditional generalized linear models (GLM) Poisson regression model were compared for optimal analysis.

**Results:**

Compared to that of the GLM Poisson regression model, a significantly lower corrected Akaike Information Criteria (AICc) was reported in the GWPR model (61953.0 in GLM vs. 43218.9 in GWPR). Spatial auto-correlation of residuals was not found in the GWPR model (global Moran’s I = − 0.005, *p* = 0.468), inferring the capture of the spatial auto-correlation by the GWPR model. Cities with a higher gross domestic product (GDP), limited health resources, and shorter distance to Wuhan, were at a higher risk for COVID-19. Furthermore, with the exception of some southeastern cities, as population density increased, the incidence of COVID-19 decreased.

**Conclusions:**

There are potential effects of the sociodemographic factors on the COVID-19 incidence. Moreover, our findings and methodology could guide other countries by helping them understand the local transmission of COVID-19 and developing a tailored country-specific intervention strategy.

## Background

The coronavirus disease 2019 (COVID-19) pandemic, caused by the severe acute respiratory syndrome coronavirus 2 (SARS-CoV-2), began in December 2019 and has spread quickly among the population [1]. Since the outbreak of COVID-19 in Wuhan, Hubei Province, Chinese government has taken unprecedented measures in response to the serious public health issue [2]. Since then, the COVID-19 epidemic in China has been basically brought under control, with a total of 80,744 confirmed cases as of March 25th, 2020 [3], after which almost all of the newly confirmed cases are the imported cases from abroad. Still, COVID-19’s impact is global, with approximately 29 million confirmed cases and over 820,000 deaths among 188 countries by the end of September 14th, 2020 [4]. Therefore, to prevent and control the pandemic, it is crucial to study the features and risk factors for COVID-19. Although numerous researchers have already conducted studies on the epidemiological characteristics, clinical diagnosis and treatment methods for COVID-19 [5–9], few have reported on the geographical distribution of COVID-19 in relation to the sociodemographic factors of different regions.

In medical research, most studies utilize conventional regression models, such as ordinary least square regression and generalized linear models (GLM) [10–12]. However, these conventional regression models generate bias by producing average parameters over the whole studied regions without considering the potential geographical variation. Geographically weighted regression (GWR) is a powerful approach to explore the possible geographical variations of mortality and incidence of infectious diseases and other health problems across space [13, 14]. The geographically weighted Poisson regression (GWPR), extended from GWR, was initially developed to model small-scale mortality that followed the Poisson distribution. Recently, GWPR is increasingly used to explore the relationships between the incidence or mortality of diseases and geographically changing factors [15–18].

Therefore, the main issues addressed in this study are as follows: a) to describe the geographical characteristics of COVID-19 incidence across different cities in China; b) to explore the spatially varying relationship of COVID-19 incidence to distances to Wuhan, GDP, health resources, and population density.

## Methods

### Data sources and data setting

Using the available surveillance data on COVID-19 in China, we conducted a geographic epidemiological study with the city as the basic geographical unit. Data on the confirmed cases of COVID-19 as of March 25th, 2020 in each city was extracted from reports of the National Health Commission of the People’s Republic of China and Provincial health committees [3]. From the 2019 China Statistical Yearbooks [19], we also extracted data on the gross domestic product (GDP), population of inhabitants, land area, and health resources indicators (including number of health personnel per 1000 people, number of hospital beds per 1000 people, and number of health institutions per 1000 people) in each city of China.

Each city’s population density was calculated by dividing the population of inhabitants per year by local land area. There is a 3-level administrative structure in China, consisting of the province, city, and district/county. According to the Ministry of Civil Affairs of the People’s Republic of China, we divided China into 346 cities. However, due to the lack of information on COVID-19 in Hong Kong, Macau, Taiwan, and Dongsha Islands, only 342 cities were incorporated in the analysis.

The study’s basic geographic unit was cities, of which the geographic location was defined as the geographic coordinates ((i.e., latitude/longitude) of the city center where the governmental agencies locates. The geographic information on China cities, including the longitude, latitude, and distance to Wuhan, was acquired from the Google Earth (https://www.google.com/earth/).

### Data analyses

The incidence of COVID-19 in each city was measured as the number of confirmed cases per million people. A principal component analysis was performed to extract a synthesized variable by using software SPSS 20.0 with three indicators related to health resources, including the number of health personnel per 1000 people, number of hospital beds per 1000 people, and number of health institutions per 1000 people. The Kaiser-Meyer-Olkin value and Bartlett’s test of sphericity were used to evaluate the reliability of principal component analysis. In the study, the Kaiser-Meyer-Olkin value was 0.665 and the *P*-value of Bartlett’s test of sphericity was < 0.001. The first principal component with a variance contribution of 81.12%, was adopted to represent the comprehensive conditions of health resources for different cities in China. GDP was used as a proxy for the socioeconomic status of each studied city. The synthesized health resources variable, GDP, population density as well as distance to Wuhan of each city were defined as explanatory variables in this study. The ArcGIS 10.2 software [20] (Environmental Systems Research Institute, Inc., Redlands, CA, US) was used to map the geographic distributions of COVID-19 incidence and explanatory variables by city. Patients’ identification number, area codes as well as research variables (such as incidence, GDP, and population density) were input in Excel software as data, sorted and further imported into ArcGIS. The area codes were obtained from the database of the Regulation of the Ministry of Civil Affairs of the People’s Republic of China, then the data table was linked to the map file using the area codes to draw the visual map.

The traditional GLM Poisson regression analysis was performed by R 3.5.3 software based on the assumption that the COVID-19 incidence follows the Poisson distribution. The fitting formula of the analysis is expressed as

$$ {\mathrm{lnO}}_{\mathrm{i}}={\upbeta}_0+{\upbeta}_1\left(\mathrm{DEN}\right)+{\upbeta}_2\left(\mathrm{GDP}\right)+{\upbeta}_3\left(\mathrm{DIST}\right)+{\upbeta}_4\left(\mathrm{HEA}\right)+{\upvarepsilon}_{\mathrm{i}} $$

where O_i_ denotes the incidence of COVID-19 in city i, β_0_ is the global intercept, β_j_ (j = 1,2,3,4) are model parameters corresponding to explanatory variables. DEN is the average population density (100 inhabitants/km^2^) of city i. GDP is the gross domestic product (100 million Renminbi Yuan) of city i. DIST is the straight-line distance (100 km) of the municipal building of city i to the municipal building of Wuhan. HEA is the synthesized variable obtained through principal component analysis to reflect health resources of city i, and ε_i_ is the error term of city i.

In the GWPR model, coefficient changes with geographic locations, which means the GWPR model can capture the spatial data’s instability and find the local association between the dependent variable and explanatory variables. The formula of the GWPR model is expressed as

$$ {\mathrm{lnO}}_{\mathrm{i}}={\upbeta}_0\left({\mathrm{u}}_{\mathrm{i}},{\mathrm{v}}_{\mathrm{i}}\right)+{\upbeta}_1\left({\mathrm{u}}_{\mathrm{i}},{\mathrm{v}}_{\mathrm{i}}\right)\ \left(\mathrm{DEN}\right)+{\upbeta}_2\left({\mathrm{u}}_{\mathrm{i}},{\mathrm{v}}_{\mathrm{i}}\right)\ \left(\mathrm{GDP}\right)+{\upbeta}_3\left({\mathrm{u}}_{\mathrm{i}},{\mathrm{v}}_{\mathrm{i}}\right)\ \left(\mathrm{DIST}\right)+{\upbeta}_4\left({\mathrm{u}}_{\mathrm{i}},{\mathrm{v}}_{\mathrm{i}}\right)\ \left(\mathrm{HEA}\right)+{\upvarepsilon}_{\mathrm{i}} $$

where (u_i_,v_i_) denotes the two-dimensional coordinates of each city, and the definitions of other model parameters are similar to those in the GLM Poisson regression model mentioned above. The GWR 4.0 software (https://gwr4.software.informer.com/download) was used to calibrate the GWPR model with the iterative reweighted least-squares method. A distance-based weighting scheme was used to allocate weights to each city by taking samples within a defined neighbourhood into calculation and by giving more weights to nearby samples than faraway samples. The kernel type and function for geographic weighting to estimate local coefficients for each city and bandwidth size was adaptive bisquare. The best bandwidth size was determined automatically using the golden section search method, based on the lowest corrected Akaike Information Criteria (AICc). Because the spatial auto-correlation is an important issue in the GLM Poisson regression model, each observation’s spatial auto-correlation is therefore expected to be removed after adjusting for the non-stationary effect in the GWPR model. To assess the spatial auto-correlation of both the GLM Poisson regression model and the GWPR model, Moran’s I coefficient, which ranges from − 1 to 1 [21], was used. When Moran’s I equals to zero, it signifies no spatial auto-correlation. In this study, the AICc and Moran’s I coefficient were used to measure how good the fit of the GWPR model and GLM is.

The complete analysis is as follows. Firstly, a traditional GLM Poisson regression analysis was performed using R 3.5.3 software, to estimate the effects of explanatory variables on COVID-19 incidence in China’s 342 cities. Considering that spatial auto-correlation might not be adjusted by the traditional GLM Poisson regression model, all explanatory variables were taken into the GWPR model in the GWR 4.0 software to explore the geographical disparities in the effects of both independent and dependent variables. Lastly, ArcGIS 10.2 software was used to display the distribution of the COVID-19 incidence and sociodemographic factors on the map of China and to intuitively reflect the geographical differences in the relationship between sociodemographic factors and COVID-19 incidence.

## Results

**Fig. 1:**
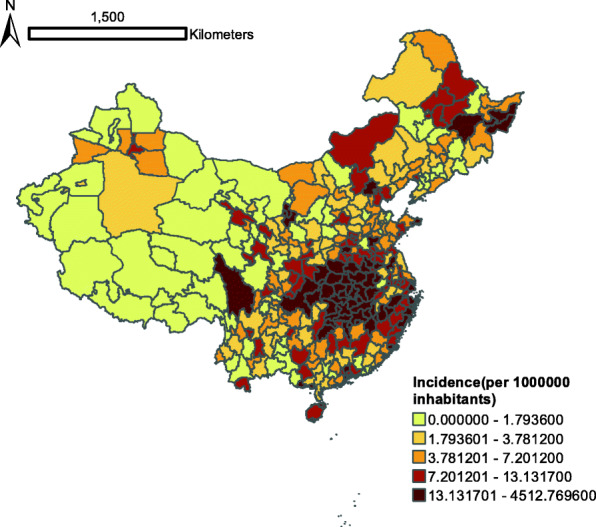
Spatial distribution of the COVID-19 incidence in China

By March 25th, 2020, 80,744 confirmed cases of COVID-19 were diagnosed in 342 cities across China, with an incidence of 57.9 per million. Among the studied cities, Wuhan has the highest incidence of COVID-19 (4512.8/1000000 people), while some cities in the west have the lowest incidence (few confirmed cases) (Shown in Fig. 1). The top ten cities with the highest incidence are shown in Table 1. According to the global Moran’s I statistic (Moran’s I = 0.039, *p* < 0.05), the incidence of COVID-19 had positive auto-correlation or clustered patterns all over China.

**Table 1:** Summary of top ten cities with the highest incidence

Area	Confirmed cases	Incidence(1/1000000 inhabitants)	GDP(100 million Renminbi Yuan)	Population density(100 inhabitants/km^2^)	Health resources	Distance(100 km)
Wuhan City	50,006	4512.7696	14847.29	12.9315	1.9202	0
Ezhou City	1394	1293.4950	1005.3	6.7525	−0.9887	0.6075
Xiaogan City	3518	715.0407	1912.895	5.5256	−0.2475	0.5229
Suizhou City	1307	589.6152	1011.185	2.3004	−0.7336	1.5029
Huanggang City	2907	459.2417	2035.203	3.6261	0.2521	0.5647
Huangshi City	1015	410.8147	1587.333	5.391	−0.6438	0.8283
Xianning City	836	328.7068	1362.417	2.608	−0.6501	0.8362
Jingmen City	928	320.3867	1847.89	2.3351	−0.4865	2.0705
Jingzhou City	1580	282.6375	2082.184	3.9249	−0.0049	2.0005
Yichang city	931	225.1049	4064.181	1.Xie J, Tong Z, Guan X, Du B, Qiu H. Clinical Characteristics of Patients Who Died9481	−0.1021	2.8901

**Fig. 2:**
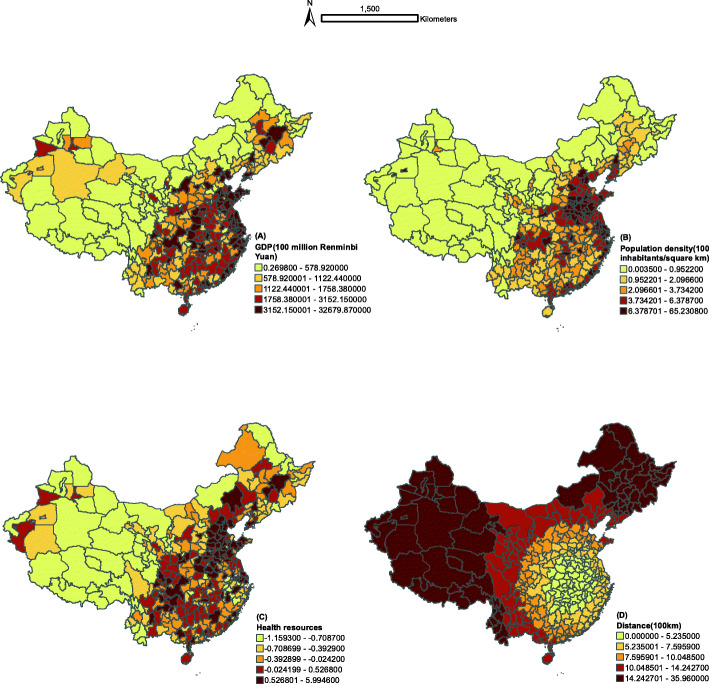
Spatial distribution of the exploratory variables in China

Considerable geographical disparities were found in the distribution of our explanatory variables among the studied cities. Compared with the western cities, China’s central and eastern cities have a higher socioeconomic standing (Fig. 2a), denser population (Fig. 2b) and better health resources (Fig. 2c). The distance between Wuhan and each studied city is presented in Fig. 2d. A more detailed description of these study variables is provided in Table 2.

**Table 2:** Summary of descriptive statistics of the independent variables and dependent variables

	Confirmed cases	Incidence(1/1000000 inhabitants)	GDP(100 million Renminbi Yuan)	Population density(100 inhabitants/km^2^)	Health resources	Distance(100 km)
Min	0	0	0.2698	0.0035	−1.1593	0
X_25%_	6	2.2616	689.1571	1.2439	−0.6445	5.5985
Median	17	5.1335	1368.0092	2.7069	−0.2368	8.5439
X_75%_	47	10.7601	2704.9100	5.7640	0.2907	13.0946
Max	50006	4512.7696	32679.8700	65.2308	5.9946	35.9600

*Min* minimum value, *X*_*25%*_ first quantile, *X*_*75%*_ third quantile, *Max* maximum value

The GLM Poisson regression model shows that the intercept and four explanatory variables are all at a significant level of 1% (Table 3). The distance of each studied city to Wuhan is negatively associated with the incidence of COVID-19. When the distance increases by 100 km, the incidence of COVID-19 decreases approximately by a factor of 0.7818. Furthermore, local population density and health resources in each city also show an inverse correlation with the incidence of COVID-19, suggesting that higher population density and better health resources might reduce the incidence of COVID-19. Interestingly, a higher GDP is associated with an increased incidence, although the correlation is very weak (the coefficient is 0.0002). After controlling for all explanatory variables using the GLM Poisson regression model, residuals still exhibit positive spatial auto-correlation (global Moran’s I = 0.128, *p* < 0.001), indicating that GLM Poisson analysis is inadequate to address the non-stationary spatial relationships.

**Table 3:** Summary statistics of traditional GLM Poisson regression model

Variable	Coefficient	Standard Error	Z-value	*p*-value
Intercept	1.7903	0.0076	234.6176	< 0.001
GDP	0.0002	0.000002	106.9084	< 0.001
Population density	−0.0373	0.0013	−29.2232	< 0.001
Health resources	−0.3770	0.0087	−43.4426	< 0.001
Distance	−0.7818	0.0017	− 462.6279	< 0.001

Corrected Aikake information criterion (AICc): 61953.0

Further fitting GWPR model with spatially varying intercept and explanatory variables (Table 4) found a significantly lower AICc than fitting GLM Poisson regression model (43,218.9 in GWPR vs. 61,953.0 in GLM, respectively). No spatial auto-correlation of residuals was found in the model (global Moran’s I = − 0.005, *p* = 0.468), inferring that the spatial auto-correlation had been captured by the GWPR model.

**Table 4:** Summary statistics of local GWPR model

Variable	Minimum	Mean	Standard Deviation	Maximum
Intercept	1.4348	1.7321	0.1577	2.0355
GDP	0.000198	0.000235	0.000021	0.000293
Population density	−0.1095	−0.0411	0.0415	0.0260
Health resources	−0.8335	−0.5141	0.1696	−0.1046
Distance	−1.0596	−0.8139	0.1010	−0.6655

Corrected Aikake information criterion (AICc): 43218.9

**Fig. 3:**
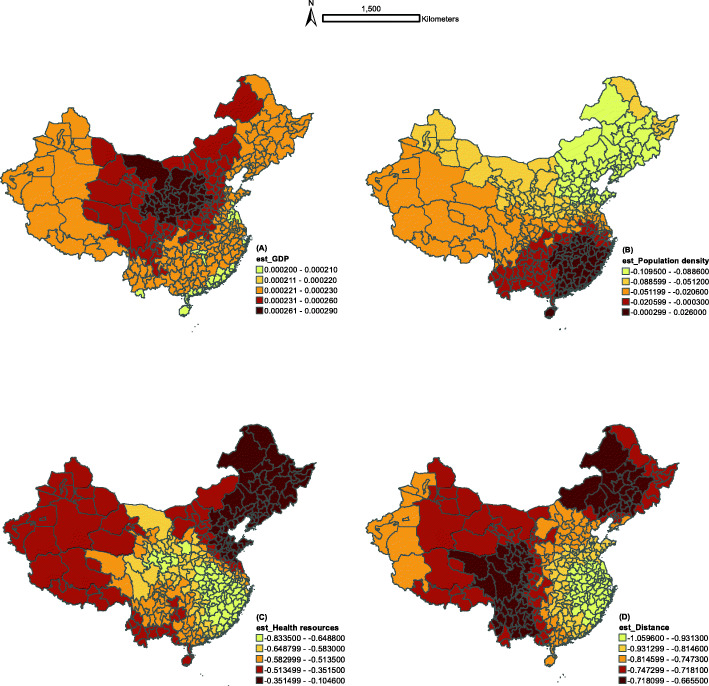
Spatial distribution of the coefficients of exploratory variables in the GWPR model

Figure 3 shows the spatial varying coefficients of four explanatory variables in the GWPR model. The economic indicator GDP is positively associated with the incidence of COVID-19, with higher coefficients in the central and northern cities (Fig. 3a). As population density increases, the incidence of COVID-19 for most of the cities decreases with exception of the southeastern cities (Fig. 3b). Health resources also have a negative impact on the incidence of COVID-19, with higher coefficients in the central and eastern cities and lower coefficients in the western and northeastern cities (Fig. 3c). A higher distance between Wuhan and the studied cities might decrease the risk of COVID-19, with the coefficient ranging from − 1.0596 to − 0.6655 among different cities (Fig. 3d).

## Discussion

To explore the potential risk factors of COVID-19, GIS (Geographic Information System) was used to visualize the geographic distributions of COVID-19 incidence in relation to the sociodemographic factors including GDP, population density, distance to Wuhan, and health resources. In this study, the local GWPR model and traditional GLM Poisson regression model were compared to find the optimal fitting model for exploring the association between the sociodemographic factors and COVID-19 incidence. The results revealed that compared with the GLM Poisson regression model, calibration of the GWPR model obviously results in an improved model fitting.

According to the GLM Poisson regression model and the GWPR model, cities with a higher GDP might have an increased risk for COVID-19. A recent study found that the rapid spread of COVID-19 worldwide tended to appear first in the most economically developed regions where high-level international trade and commercial activities were prevalent. Following the initial spread of COVID-19 along international trade routes between the developed regions, the virus spreads later to the developing areas [22]. In our study, a higher coefficient was observed in the midlands and northern cities than in the southern cities of China in the GWPR model. A possible explanation for this phenomenon is that the southern cities have more robust economy than the northern cities. The economic improvement might exert a more extensive and significant influence on the northern cities, it accordingly increase the infection density of COVID-19 [23]. Further investigation is required for more detailed causes.

Our result also revealed that with the distance to Wuhan increasing, the incidence of COVID-19 decreases among all of the studied cities based on both GLM Poisson regression model and GWPR model. The spatial varying coefficients shows a decreasing trend from the southeast to the northwest in the GWPR model. Since more than 5 million people had already left Wuhan before it was officially sealing off, we were unable to track where exactly these people had gone. Therefore, the distance to Wuhan could be used in part to represent this massive human migration. Obviously, cities located at a greater distance to Wuhan will experience less or even no contact with the infectious sources, which hinders the spread of COVID-19. On the contrary, in cities near Wuhan with convenient transportation system and a high degree of trafficking, their residents were more likely to contact with the infectious sources, which will promote the spread of COVID-19. Consistent with our current and previous findings [24], other studies have also revealed the aggregation characteristics of the virus and reminded us the importance of shutdown of the epidemic areas and isolation of the infectious sources [25].

According to the GWPR model, the coefficients of health resources were negative in 342 cities and showed a degressive trend from the southeast to the northwest, indicating that better health resources might mitigate the spread of COVID-19. Better health resources could help identify the sources of infection and enable suspected patients and close contacts to gain better access to quarantine measures, which in turn prevents the spread of COVID-19 and reduce it’s incidence. Other studies have also emphasized the importance of controlling the sources of infection and cutting off the routes of transmission [26]. However, it is worth noting that health resources were more lacking in the western cities than in the central and eastern cities of China. Previous reports have also confirmed the substantial regional disparities of both availability and accessibility to health resources in China [27]. Fortunately, since the outbreak of COVID-19, Chinese government has undertaken tremendous efforts in constructing new medical facilities, mobilizing the country’s vast and robust medical forces and accelerating the delivery of medical supplies, and as a consequence, has quickly brought the epidemic under control. This concurs with our findings. In order to effectively control the spread of COVID-19, we urge all governments to ramp up the amount of available and accessible medical and health resources in various regions. China’s situation could provide a guide to other countries on how to prepare for possible local outbreaks, especially for resource-limited countries [28].

With regard to the population density, both GWPR model and GLM Poisson regression model showed a negative association between population density of each city and the incidence of COVID-19. In the GWPR model, this effect decreases from the north, which has a lower population density, to the south, which has a higher population density. Interestingly, in paradox, COVID-19 incidence is higher in cities with a lower population density. This unique virus spreading pattern in China is possibly due to the following reasons: First, many usually highly populated large cities are much less populated during the Spring Festival in China due to massive migration of people from highly populated large cities to less populated medium and small cities as well as rural areas for the sake of family reunion. Second, after the outbreak of COVID-19 in Wuhan, many residents of highly populated large cities, including Wuhan, undertake “evasive activity” to return to less populated small cities or rural areas. Notably, a study from the United State reported that household size, rather than overall population density, is more strongly associated with the prevalence of COVID-19 [29]. Moreover, another study considered that the population density is a more useful predictor of COVID-19 infections and mortality for metropolitan areas, but not for rural areas [30]. Thus, it is necessary to deeply explore the relationship of population density to the incidence of COVID-19.

To be noted, this study has some limitations. First, the observed differences may be subject to many unobserved and unavailable confounding factors such as age, gender, nationality, and other natural factors, all of which were not accounted in the multivariate analysis. Second, because this study is based on surveillance data, the causal relationship between sociodemographic characteristics and the incidence of COVID-19 could not be demonstrated. Third, due to different policies and measures in response to COVID-19 in each country, our results could not be extrapolated to other countries. Nevertheless, to the best of our knowledge, this study is the first to combine the COVID-19 surveillance and sociodemographic data into GIS and analyze the possible risk factors of COVID-19 incidence in China from the spatial perspective, filling the gap of knowledge of this geographical region.

## Conclusions

Our results show that local GWPR model is a better fitting model to investigate the effects of sociodemographic factors on COVID-19 than the traditional GLM Poisson regression model. Cities with a higher GDP, limited health resources, and a shorter distance to Wuhan, were at a higher risk for COVID-19. Moreover, the relationship between the population density and COVID-19 incidence might be mediated by the peculiar set of circumstances during the spread of the virus in China, i.e., the Spring Festival and Spring Transportation in China. In conclusion, these findings shed light on the effect of sociodemographic factors on COVID-19 incidence from the geographic perspective and have important public health policy implications for COVID-19 management and prevention in China. In addition, the study could be used as a guide for other countries to understand the local spread of COVID-19.

## Data Availability

The city-level COVID-19 confirmed case number information was made available from the Health Commission of the People’s Republic of China and Provincial health committees [3]. Data on the GDP, population of inhabitants, land area, and health resources indicators in each city of China were available from the 2019 China Statistical Yearbooks [19].

## Abbreviations

COVID-19: Coronavirus disease 2019
SARS-CoV-2: Severe acute respiratory syndrome coronavirus 2
GLM: Generalized linear models
GWR: Geographically weighted regression
GWPR: Geographically weighted Poisson regression
GDP: Gross domestic product
AICc: Corrected Akaike Information Criteria
